# Utilizing the Switching Stochasticity of HfO_2_/TiO_x_-Based ReRAM Devices and the Concept of Multiple Device Synapses for the Classification of Overlapping and Noisy Patterns

**DOI:** 10.3389/fnins.2021.661856

**Published:** 2021-06-07

**Authors:** Christopher Bengel, Felix Cüppers, Melika Payvand, Regina Dittmann, Rainer Waser, Susanne Hoffmann-Eifert, Stephan Menzel

**Affiliations:** ^1^Institute of Materials in Electrical Engineering and Information Technology II and Jülich Aachen Research Alliance (JARA)-Fit, Rheinisch-Westfälische Technische Hochschule (RWTH) Aachen University, Aachen, Germany; ^2^Peter Grünberg Institute (PGI 7 & 10), Forschungszentrum Jülich GmbH and JARA-Fit, Jülich, Germany; ^3^Institute of Neuroinformatics, Eidgenössische Technische Hochschule (ETH) Zurich, Zurich, Switzerland

**Keywords:** memristor, ReRAM, synapse, stochastic, signal processing, pattern classification

## Abstract

With the arrival of the Internet of Things (IoT) and the challenges arising from Big Data, neuromorphic chip concepts are seen as key solutions for coping with the massive amount of unstructured data streams by moving the computation closer to the sensors, the so-called “edge computing.” Augmenting these chips with emerging memory technologies enables these edge devices with non-volatile and adaptive properties which are desirable for low power and online learning operations. However, an energy- and area-efficient realization of these systems requires disruptive hardware changes. Memristor-based solutions for these concepts are in the focus of research and industry due to their low-power and high-density online learning potential. Specifically, the filamentary-type valence change mechanism (VCM memories) have shown to be a promising candidate In consequence, physical models capturing a broad spectrum of experimentally observed features such as the pronounced cycle-to-cycle (c2c) and device-to-device (d2d) variability are required for accurate evaluation of the proposed concepts. In this study, we present an in-depth experimental analysis of d2d and c2c variability of filamentary-type bipolar switching HfO_2_/TiO_x_ nano-sized crossbar devices and match the experimentally observed variabilities to our physically motivated JART VCM compact model. Based on this approach, we evaluate the concept of parallel operation of devices as a synapse both experimentally and theoretically. These parallel synapses form a synaptic array which is at the core of neuromorphic chips. We exploit the c2c variability of these devices for stochastic online learning which has shown to increase the effective bit precision of the devices. Finally, we demonstrate that stochastic switching features for a pattern classification task that can be employed in an online learning neural network.

## Introduction

In the past years, conventional computers have experienced an enormous rise in computational power and now exceed the human capabilities for tasks like mathematical calculations, or even games like Go (Silver et al., 2016) and StarCraft II (Vinyals et al., 2019). However, severe power consumption limitations stand in the way of realizing complex tasks like navigation and recognition in conventional hardware. Solutions for such tasks are much faster and multiple orders of magnitude more energy-efficiently accomplished by biological systems like the human brain. With the rise and accompanying promises of machine learning, humans are now trying to build systems that can energy-efficiently solve the kind of problems that biological systems are efficient at. These tasks have several common attributes such as noisy inputs and ambiguous rules. While a task like playing chess can be formalized in a way that allows a computer to outperform based on brute force, i.e., by being able to test millions of positions this is not true for complex tasks, where the space of possible solutions explodes far earlier. One approach toward enabling computers to cope with this unconventional challenge is neuromorphic computing in which physical phenomena are used as computational primitive and where signals are represented in an analog form (Mead, 1990). This approach might be facilitated by the use of new memristive devices based on spin (Magnetic Random Access Memory—MRAM) (Apalkov et al., 2016), phase change (Phase Change Memory—PCM) (Wong et al., 2010) or redox reactions (Redox based Resistive Random Access Memory—ReRAM) (Waser et al., 2009). These memristive systems are usually structured in a matrix-like fashion to realize neuromorphic computing systems (Steinbuch, 1961, 1963; Ziegler et al., 2020). Here, ReRAM devices offer several benefits: their low-power, dense integration feasibility and rich device physics open up the opportunity for online learning. A hybrid CMOS-ReRAM neuromorphic chip can serve as an adaptive substrate, accelerating complex problem solving while maintaining a low power budget. Employed at the edge, these hybrid systems can be used as a first data processing stage by gathering and then filtering raw sensor outputs. These ideally autonomous edge systems will often require online learning capabilities to adapt to their environment and to mitigate aging effects of the single components. As these systems will be severely power limited, efficient and finely tunable synapses will be necessary. Since synapses are one of the most important elements in neuromorphic computing systems, any concrete implementation of synaptic elements using, for example, emerging device technology will need to be evaluated with regard to the synapses dynamic range (bounds of the synaptic efficacy) and their grade of tunability (analog depth of the synaptic efficacy) (Giulioni et al., 2009). The utilized filamentary-type ReRAM devices rely on the movement of oxygen vacancies in an insulating matrix for their resistance change. Based on this physical mechanism two pathways have emerged to use them as synapses. The first one is focused on the analog operation of the devices. In this approach, incremental reconfiguration of these mobile vacancies is the goal, leading to gradual resistance changes in a limited conductance window (Prezioso et al., 2015; Covi et al., 2016; Frascaroli et al., 2018). It has been shown, however, that expanding this conductance window diminishes the possibility for such incremental changes and more abrupt, self-accelerating switching phenomena dominate (Fleck et al., 2016; Cüppers et al., 2019). The second operation possibility is based on using them as binary switches and exploiting their stochastic switching behavior. Especially for the filamentary-type valence change mechanism (VCM) devices this operation mode must be seen under the aspect of device-to-device (d2d) and cycle-to-cycle (c2c) variability. This is the focus of this work.

Several approaches have been exploited for synapse emulation utilizing the stochastic nature of the resistance change in ReRAM devices. From the literature, two main pathways are identified, these are the single-device and the compound-device architectures. On the side of single device architectures, extensive studies exist on the variability phenomenon both for d2d and c2c aspects (Jo et al., 2009; Suri et al., 2013; Yu et al., 2013a; Naous et al., 2016; Wenger et al., 2019; Zahari et al., 2020). The idea to incorporate multiple resistive devices into a synapse has been introduced earlier (Gaba et al., 2013; Bill and Legenstein, 2014; Hu et al., 2014; Singha et al., 2014; Boybat et al., 2018, 2019; Payvand et al., 2018, 2019). All variants followed the strategy to compensate for the short-comings of single devices by forming compound synapses. Singha et al. (2014) concluded that a synapse construction based on multiple parallel devices does not yield a significant advantage over single analog switches, but can be chosen as an alternative pathway when other tradeoffs emerge. However, while extracting the typical switching voltage stochasticity from c2c, their work doesn’t consider the aspect of d2d variability, which should yield a significantly different result for a single, analog device synapse compared to the multi-device approach (Singha et al., 2014). Boybat et al. employed parallel PCM devices as analog synapses for three different neural network tasks. They conducted a very detailed study of variability between individual devices and the switching stochasticity over multiple cycles. However, the main goal of their work was to stabilize analog conductance changes in the synapse’s update under application of repeated current pulses (Boybat et al., 2018).

Common for previous works published on the subject of stochastic switching of ReRAM devices in neural networks is the utilization of behavioral models. These lead to voltage-dependent switching probability models such as the Poisson distribution (Jo et al., 2009; Gaba et al., 2013; Naous et al., 2016; Payvand et al., 2018; Zahari et al., 2020), sigmoidal distribution (Wenger et al., 2019), Gaussian distribution (Yu et al., 2013b) and lognormal distribution (Medeiros-Ribeiro et al., 2011; Hu et al., 2014), and even linear dependence (Singha et al., 2014). By definition, these models only capture the minimal required behavioral aspects and possess little to no predictive character for any setup modification. However, with the aim to correlate the single ReRAM device behavior with the neuromorphic circuit behavior, it is imperative to use more detailed compact models that are able to capture the full dynamical spectrum of the employed device type.

In this work, we follow this comprehensive approach. Therefore, we combine detailed experimental characterization of d2d and c2c variability of stochastically switching HfO_2_/TiO_x_ nano-sized VCM devices with a physically motivated compact model. The latter describes a broad spectrum of switching behavior as well as the variability phenomena that are observed in experiment. Going further, the parallel operation of multiple devices in the proposed synaptic building block is experimentally demonstrated and the physical origin underlying the benefit of increasing the number of devices is revealed. Through a theoretical analysis the influence of various variability components on the synapse behavior is disentangled and evaluation criteria for a favorable synapse behavior are developed.

We then demonstrate that network performance issues, caused by the use of devices under the impact of d2d and c2c variability can be resolved by employing the multi-device synapses. A spiking neural network (SNN) for the classification of overlapping patterns served as a benchmark in this regard. Its resilience toward pattern overlap and noisy input signals is demonstrated. The algorithm employed for training this network, a technologically plausible adaptation of the Delta rule, uses stochastic rounding (Hopkins et al., 2020) as a means to improve the network’s performance. To improve the network’s behavior a novel hyperparameter tuning algorithm is established which can deal with the device variability.

In the following section, we describe the used materials and experimental methods as well as the simulation model and the network we used. The subsequent results’ section is split into four parts. The first part focuses on the statistical device variability characterization and the modeling of the SET process on the 1 μs timescale which is the timescale of the considered application. The second part verifies that the employed model captures the device switching dynamics over multiple orders of magnitude in switching time, including the c2c behavior. The third part shows our findings on the basic synaptic building block of the neural network that is formed by a parallel connection of ReRAM cells. The fourth part shows the performance of the SNN utilizing stochastic gradient descent for very noisy pattern detection. Following this, we discuss our results and finalize with proposing future strategies for the optimization of the SNN.

## Materials and Methods

### Pt/HfO_2_/TiO_x_/Ti Nano-Crossbar Devices

In this study, nano-sized Pt/HfO_2_/TiO_x_/Ti crossbar devices with lateral dimensions of 100 nm by 100 nm were used. First, the 25 nm thick sputtered Pt bottom electrode (BE) is patterned by electron beam lithography on top of a 5 nm Ta adhesive layer, a 10 nm SiO_2_ insulating layer and a Si substrate. The BE is then structured by back-etching using Reactive Ion Beam Etching (RIBE). Subsequently, the 3 nm HfO_2_ and the 3 nm TiO_x_ layers are deposited via plasma-enhanced atomic layer deposition (ALD) and thermal ALD, respectively. The 10 nm Ti top electrode (TE) metal and a 20 nm Pt protective layer are deposited by electron beam evaporation. The second electron beam lithography defines the structure of the TE. The protective Pt layer, the Ti TE and the oxide layers are finally back-etched via a RIBE process. Details about the fabrication process are given in (Hardtdegen et al., 2018).

Our previous studies employed this system for a description of enhanced switching performance by insertion of intentionally grown TiO_x_ at the HfO_2_/Ti interface (Hardtdegen et al., 2018), the underlying physical mechanism of gradual and abrupt switching (Cüppers et al., 2019), and the switching variability phenomenon inherent to all filamentary VCM-type switches (Bengel et al., 2020). It has been shown, that the described stack can be utilized for all typical memristor measurement techniques. Hence, this study also employs the same stack as a test vehicle for the proposed neuromorphic functionality.

### Electrical Measurements

Before showing typical resistive switching an electroforming step of the as-prepared devices has to be performed. It is carried out by applying a negative polarity voltage sweep with an Agilent B1500A semiconductor analyzer with a measurement-sided current compliance of 50 μA and a sweep rate of 0.66 V/s. The electroforming voltage of the devices is at (-2.7 ± 0.1) V, applied to the active electrode (AE). The device switching performance is assured by measuring 30 consecutive voltage sweeps at a low sweep rate of 0.67 V/s. Typically, a high device yield of more than 90% is achieved by this method.

#### Individually Contacted Devices

In this study, the ReRAM devices were operated in two different ways. Employed as single devices, they were contacted by two probe needles, which were placed close to the device under test to minimize parasitic effects caused by series resistances and capacitances.

For the SET pulse measurements on single devices over multiple orders of magnitude in switching time, a custom-built pulse measurement setup comprising an Agilent B1110A pulse-/pattern generator and a Tektronix TDS6804B digital storage oscilloscope is used. For the exact measurement setup, the reader is referred to Marchewka et al. (2016). Furthermore, a Keithley 4200 SCS semiconductor analyzer was employed for the transient voltage-current measurements. For single device measurements that only required read-pulse-read operation, an ArC ONE platform of ArC Instruments Ltd. was employed.

#### Probe Card Arrangement

The second contacting scheme exploited the device arrangement consisting of arrays of 32 × 1 devices connected in parallel. Specifically, the bottom electrodes of these devices are connected and a common ground contact is added on one side of this 32 × 1 line array. The devices are hence contacted individually on their respective 32 top electrodes with a line probe card, while a 33rd probe is placed on the ground contact. This allows for parallel signal application.

In this context, the capability of the ArC ONE of routing the signals to multiple electrodes simultaneously (termed *MultiBias module* by the manufacturer) is used in this study.

### JART VCM v1b Model Including Variability

**FIGURE 1:**
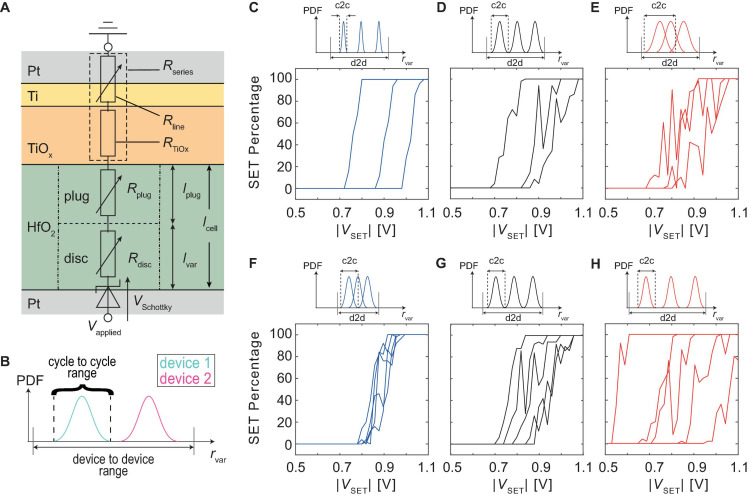
**(A)** Shows the equivalent circuit diagram of the JART VCM v1b model used to describe a Pt/HfO_2_/TiO_x_/Pt (HOTO) device. The exact stack properties can be found in Hardtdegen et al. (2018). **(B)** Schematically shows the modification made to the variability model exemplary for the variability model parameter *r*_var_. For each device, a seed parameter is drawn from the d2d range. Around this seed parameter, the cell can change its variability parameters by the smaller c2c range. **(C–E)** Show the effect of different amounts of the c2c variability on the SET probability behavior for three cells. For **(C)**, the c2c percentage was 5%, for **(D)** it was 15%, and for **(E)** it was 25%. **(F–H)** Show the effect of different amounts of d2d variability. This was achieved by decreasing the d2d range and the variation coefficient for **(F)**. For **(G)** the values from Table 2 were chosen and for **(H)** the d2d range was increased. The changes are conceptually shown by the small PDFs given on top of diagram **(C–H)**.

For the simulations the Jülich Aachen Resistive Switching Tools (JART) VCM v1b variability model is used (Cüppers et al., 2019; Bengel et al., 2020; Juelich Aachen Resistive Switching Tools [JART], 2019). It is a SPICE level, physics-based compact model describing filamentary bipolar resistive switching devices based on the valence change mechanism (VCM)-type Redox-based Resistive Random Access Memory (ReRAM). The model relates the observed resistive switching behavior to the motion of ionic defects leading to a modulation of the concentration *N*_disc_ close to the AE. A high concentration represents a low resistive state, whereas a decreased concentration leads to an increased resistance. The equivalent circuit diagram of the model is shown in Figure 1A.

The deterministic model parameters are listed in Table 1.

**TABLE 1:** Simulation parameters (for the explanation of the symbols see Bengel et al., 2020).

Symbol	Value	Symbol	Value
*l*_cell_	3 nm	A*	6.01⋅10^5^ A/(m^2^K^2^)
*l*_disc_	0.25 nm	*eΦ*_Bn0_	0.18 eV
*r*_fil_	30 nm	*eΦ*_n_	0.1 eV
*z*_vo_	2	*μ*_n_	4⋅10^–6^ m^2^/(Vs)
*a*	0.25 nm	*N*_plug_	20⋅10^26^ m^–3^
*ν*_0_	2⋅10^11^ Hz	*N*_disc, max_	0.25⋅10^26^ m^–3^
*ΔW*_A_	1.6 eV	*N*_disc, min_	0.2⋅10^23^ m^–3^
*ε*	17⋅ε_0_	*R*_series_	1,300 Ω
*ε*_Φ__B_	5.5⋅ε_0_	*R*_th, eff, SET_	4⋅10^7^ K/W
*T*_0_	293 K	*R*_th, eff, RESET_	14⋅10^6^ K/W

In our previous work (Bengel et al., 2020), d2d and c2c variability were realized by drawing a random set of parameters from a truncated Gaussian distribution (seed parameters) for d2d variability and then changing those parameters, throughout the simulation, around that seed parameter to produce c2c variability. The truncation of the Gaussian distribution determines the maximum deviation of the parameters around its mean value and was the same for the initialization as well as for the variation throughout the simulation. The variability parameters were chosen as the minimum and maximum oxygen vacancy concentration in the disc *N*_disc, min_ and *N*_disc, max_, as well as the radius of the switching filament *r*_fil_ and the length of the disc region *l*_disc_. The choice of parameters was motivated based on the experimental findings of Baeumer et al. (2017), where it was shown that the filament could form at different positions in the cell, which lead to variability in the LRS and HRS. Compact modeling aims to provide a tool for the design of circuits as well as bridging the gap between device-level technology and circuit design. Therefore, if a certain application is considered, the compact model must adequately model the device behavior under the conditions of the specific experiment. For this paper, the relevant experiment is the measurement of the SET probabilities at different applied voltages starting from a specific range of HRS. Due to the large cycling variability and the large spread between different devices observed in our experiment, we decided to modify how we use the variability model by splitting the ranges for d2d and c2c variability (see Figure 1B). In the new version, a cell is initialized by drawing the seed variability parameters from a truncated Gaussian distribution. The c2c variability is achieved by changing those parameters during the simulation. However, the range in which these parameters can change during cycling is limited independently from the range of the d2d variability through a fixed percentage around the seed value. This enables us to tune d2d and c2c variability independently to fit the model parameters to the measurements. The new implementation, therefore, uses three different parameters to modify the different variability of the model. The relative standard variation which determines the width of the truncated Gaussian distribution is used to initialize the devices. This quantity influences mostly the d2d variability since it determines whether the parameters will be initialized closer or further away from the median value on average. The c2c variability is controlled by two parameters namely the c2c percentage and the maximum step size. The c2c percentage determines the range around the drawn set of seed parameters for each device in which the parameters can change through repeated switching. On the other hand, the maximum step size determines by how much variability parameters can maximally change between two subsequent switching cycles. The influence of these different parameters can be observed in Figures 1C–H. C, D and E show the effect of different amounts of c2c variability on the SET probabilities while F, G, and H show the effect of different amounts of d2d variability. It can be observed that increasing the c2c variability makes the behavior of single devices more stochastic. This implies that increasing voltages do not always result in an increase of SET probability but might also decrease it. Increasing the d2d variability spreads the SET probability curves across a larger voltage range.

The parameters related to the variability are given in Table 2. They were kept constant throughout the whole results chapter showing the high degree of consistency between model and experiment.

**TABLE 2:** Simulation Parameters (for the explanation of the symbols see Bengel et al., 2020).

Symbol	Min/Median/Max	Symbol	Value
*N*_min, var_ [10^23^ m^–3^]	0.1/0.2/0.3	Relative standard deviation	1
*N*_max, var_ [10^26^ m^–3^]	0.05/0.25/20	c2c percentage	15%
*r*_var_ [nm]	25/30/35	Maximum stepsize	10%
*l*_var_ [nm]	0.175/0.25/0.35	–	–

### Network Fundamentals

**FIGURE 2:**
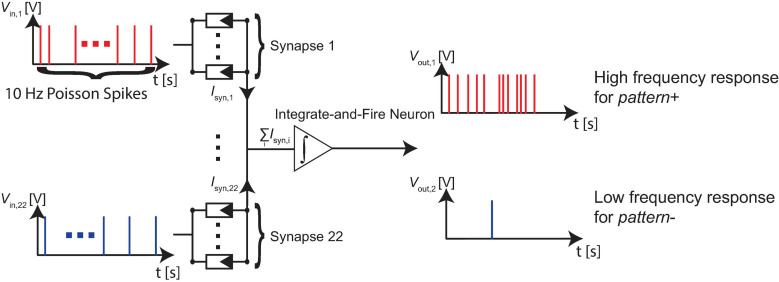
The neural network consists of 22 synapses connected to an integrate-and-fire type output neuron. The synapses consist of multiple VCM ReRAM devices connected in parallel. The studied range is between 1 and 24 cells per synapse. It is trained to react to a *pattern+* (which is presented to 11 of its synapses) with a high number of spikes and to a *pattern-* (which is also presented to 11 of its synapses) with a low number of spikes.

To highlight the power of exploiting device stochasticity, we employ our device model in a binary classification problem with overlapping features. We systematically assess the classification accuracy by raising the complexity of the problem through increasing the overlap, i.e., the mutual information between the patterns. The parameters of the variability model are drawn to initialize the required number of devices, which depends on the number of ReRAM per synapse. The devices are usually initialized with a resistive state that roughly lies between the LRS and HRS. The investigated neural network consists of 22 synapses connected to an integrate-and-fire type output neuron. The synapses each consist of one or multiple ReRAM cells connected in parallel. The studied range is between 1 and 24 cells per synapse and is realized by our VCM ReRAM model (JART VCM v1b). The network structure is shown in Figure 2.

#### Pattern Generation

**FIGURE 3:**
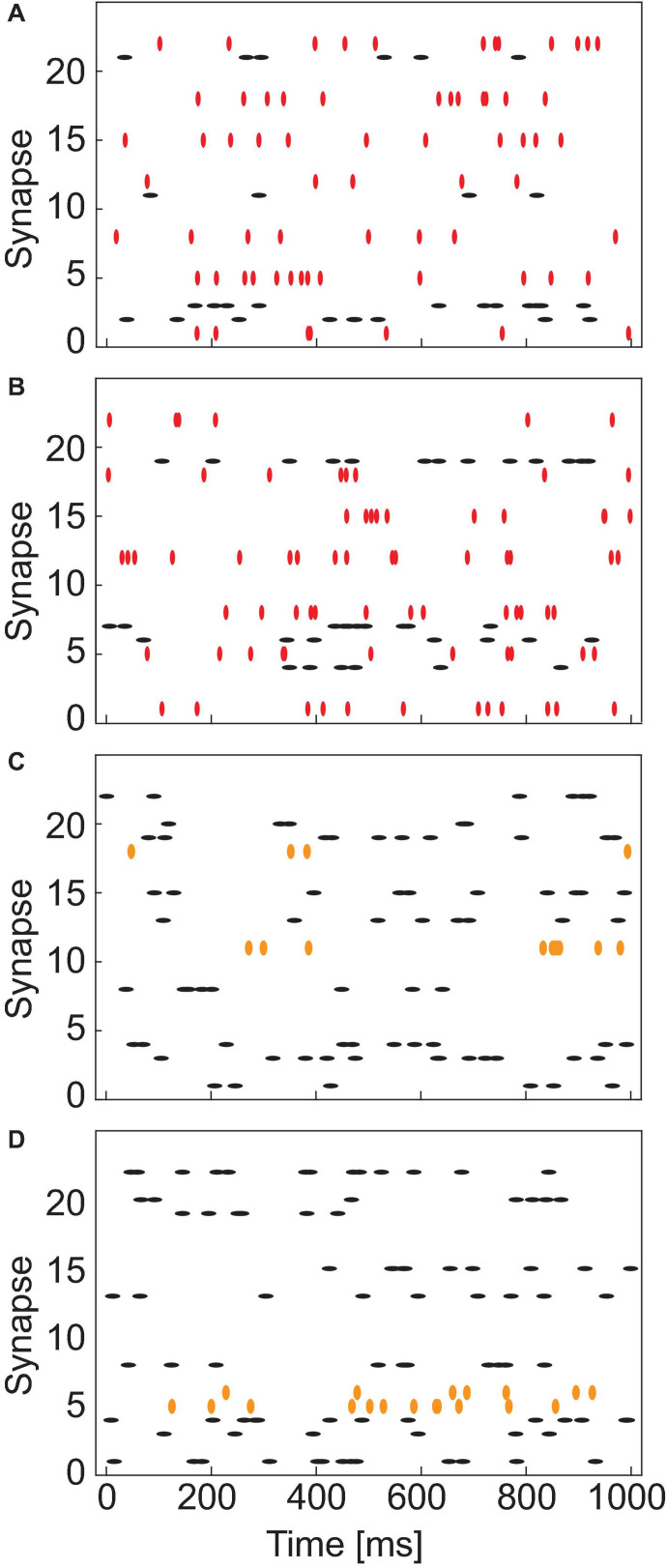
Exemplary raster plots showing the Poisson distributed spike trains which are applied to the different synapses for 1 s. **(A,B)** Show an exemplary *pattern+* and *pattern–*, respectively, with an overlap of seven between the patterns. The overlapping synapses (1, 5, 8, 12, 15, 18, 22) are marked as red vertical symbols. **(C,D)** Show two noisy *pattern+* examples. Based on the ideal pattern (not shown) two synapses were flipped (11 and 18 in **C** and 5 and 6 in **D**). The flipped synapses are marked as orange vertical symbols.

We have synthesized the patterns to have control over the complexity and to study the network accuracy as a function of the problem complexity. To generate the patterns half of the synapses are picked randomly and stimulated with a Poisson distributed spike train. The rest of the synapses are not stimulated. We call this *pattern+*. To generate *pattern*-, we define an overlapping parameter M, which is the number of common features or amount of mutual information. M number of synapses from *pattern+* are then selected randomly as the common feature of *pattern-*. The rest of the features of *pattern-* is chosen randomly from the remaining synapses that are not included in *pattern+*. The synapses making up *pattern-* are also stimulated via a Poisson distributed spike train. As an additional level of complexity, we also introduce noise in the patterns by randomly flipping a specified number of features in *pattern+* and *pattern-*. Seven noisy test patterns are generated for *pattern+* and seven for *pattern-*. These training patterns will be used during the inference phase for the evaluation of the network’s performance. A few exemplary patterns are shown as raster plots in Figure 3. Figures 3A,B show one exemplary *pattern+* and *pattern*-, respectively, with an overlap of seven. The seven overlapping patterns are marked as red vertical symbols, while the red horizontal lines represent the non-overlapping patterns. C and D show two *patterns+* in which two synapses have been flipped each. The flipped patterns are marked as orange vertical symbols, while the non-flipped patterns are marked as black horizontal symbols.

#### Training Procedure

To train the network, we utilize a technologically plausible training algorithm, namely a stochastic Delta rule algorithm, which is the simplest form of gradient descent for single-layer networks (Payvand et al., 2020). The Delta rule can be formulated as

(1) $$\Delta \,w_{i}=\lambda \cdot \left(\hat{y}-y\right)\cdot x_{i},$$

where *Δw*_i_ denotes the amount the weights that have to change in the network, λ is the learning rate which can be used to scale the amount of weight change per update, $\hat{y}$ is the target, *y* is the neuron output activity and *x*_i_ selects the synapses to which the current pattern is applied. For our binary classification problem, the target for *pattern+* and *pattern-* are the maximum and minimum firing rate of the neuron *FRMAX* and 0, respectively. Therefore, the update rule becomes

(2) $$\Delta \,w_{i}=\lambda \,\frac{F\,R\,M\,A\,X\cdot l\,a\,b\,e\,l-F\,R}{F\,R\,M\,A\,X}.x_{i},$$

where *label* is 1 when *pattern+* is applied and 0 if *pattern-* is applied. *FR* is the firing rate of the neuron in response to the applied pattern. This training rule is formalized in Algorithm 1.

**Table:** **Algorithm 1**: Delta Rule implementation with stochastic synapses.

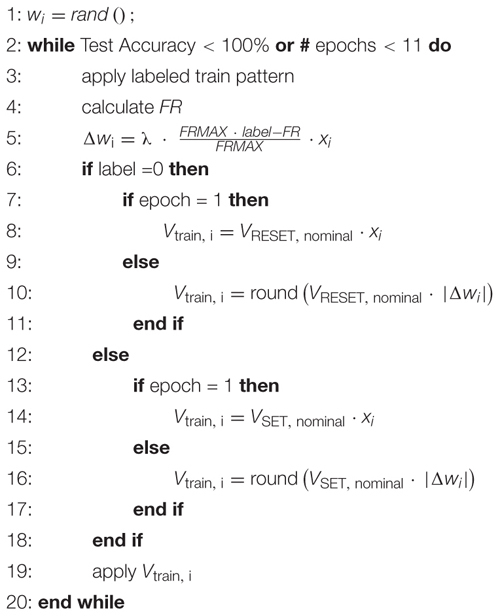

To assess the untrained accuracy all training patterns are presented to the network. At this stage, the accuracy of the network is 50% in most cases, which is the accuracy of guessing randomly. This first step is done to show that the network is trained during the next steps and starts from a bad accuracy. The simulations are performed in the following fashion. The synapses are simulated using Cadence Spectre, and the current Σ *I*_syn_ (see Figure 2) accumulated at the output node is saved. This output current is then fed into an integrate-and-fire neuron model realized in MATLAB which samples the current at 1 ms intervals. The current is summed up until the neuron threshold *I*_TH_ is reached and the integrated current is reset to zero. Each instant of time at which the neuron threshold is reached is counted as a spike of the neuron. The total number of spikes produced for 1 s is then compared with the decision threshold *FRMAX*/2. If the number of spikes is larger the pattern is interpreted as *pattern+* and if it is smaller it is interpreted as *pattern-*.

After the initial evaluation, the network is repeatedly trained and tested for 10 epochs. During the training, a noisy training pattern is applied to the specified synapses and the number of spikes *FR* is counted. This number is then compared with the target number for the current pattern which is either *FRMAX* for *pattern+* or 0 for *pattern-*. The difference between the real and wanted number of spikes is scaled by *FRMAX*, multiplied with the learning rate λ which is set to a value of 1.2 in our case, and multiplied with a vector that corresponds to the assignment of synapses of the current training pattern. This relationship is described by Eq. (2). It represents the desired weight change for all the synapses that just received a pattern. In this way, the following programming pulse will be weaker if the neuron’s response is close to the ideal response (0 or *FRMAX*) so as not to disturb the achieved weights too much, and stronger if the neuron’s response to the current pattern is far away from the ideal response. After the calculation of the distance between the ideal and the actual neuron response, the programming pulses are applied to the synapses that also received the training signals. The nominal SET (-0.8 V) and RESET (1.3 V) voltages are scaled by Δ*w*_*i*_ and rounded to the closest 100 mV increment. This scaling of the voltages modulates the switching probability of the ReRAM cells in the synapse. As the probability of switching is increased if the network’s error is higher and decreased if it is lower, this represents a technologically plausible stop learning mechanism. Additionally, this can be seen as a form of randomized or stochastic rounding (Muller and Indiveri, 2015) similar to the one implemented in Payvand et al. (2018). In previous works, stochastic rounding has been found to improve neural networks, enabling to reduce the bit size of the weights (Gupta et al., 2015) or enabling to reduce the input bit size (Gokmen et al., 2018) while keeping the accuracy constant (Gokmen et al., 2018). The scaled programming voltages are applied for 1 μs, leading to a SET/RESET of the ReRAM cells of the respective synapses. This training procedure is performed for three *pattern+* and three *pattern-* in a random succession for each epoch. After these six training rounds, the network’s performance is tested on seven test patterns for *pattern+* and *pattern-*. This procedure is repeated until the accuracy on the test pattern set reaches 100% or until 10 epochs are reached.

## Results

### Device-to-Device Variability Characterization on 1 μs Timescale

**FIGURE 4:**
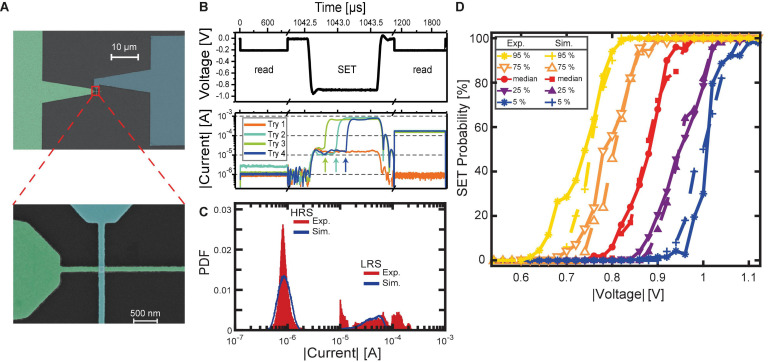
SET switching variability for 1 μs voltage stress time. **(A)** SEM picture of a single nano crossbar structure. Zoom-in to the device crossbar. **(B)** Shows a typical SET voltage stress sequence in the upper panel. A read pulse for verification of the HRS is followed by the voltage stress pulse. The resulting resistance state is detected by another read pulse. In the lower panel, possible current responses are shown in logarithmic scale. The SET transitions are indicated by the arrows. **(C)** Comparison of the read currents for HRS and LRS for simulation (blue lines) and experiment (red bars) by their respective probability density functions (PDF). **(D)** Statistics of the d2d variability for experiment and simulation.

As mentioned in section “Materials and Methods,” filamentary-type VCM devices integrated into crossbar structures as shown in the scanning electron microscope (SEM) image in Figure 4A exhibit significant d2d and c2c variability. This results in non-uniform current responses to identical voltage sequences. An example is illustrated in Figure 4B. Here, four exemplary current transients resulting from identical voltage stress are shown. The shown SET attempts were recorded on the same device in direct succession, with only a RESET to the HRS before the next SET attempt. Before the SET pulse is applied, a read signal of -0.2 V is applied to the device, confirming the HRS of the device. The following SET voltage pulse with a duration of 1 μs and an amplitude of -0.88 V causes a variable current response of the device. In the case of Try 1, the current remains at a low level for the pulse duration, exhibiting no significant increase. The subsequent read signal confirms that the device has not undergone a significant change in resistance as the current is still low. In comparison, Tries 2, 3, and 4 show an abrupt increase in absolute current during the voltage stress. As typical for filamentary VCM type devices, this transition does not occur at a deterministic time but is highly variable (*t*_delay, Try 4_ > *t*_delay, Try 2_ > *t*_delay, Try 3_). Furthermore, the current at the end of the pulse is not identical for the tries with an abrupt increase but also suffers from variability. The subsequent read signal current levels reflect the currents at the end of the SET pulse, where | *I*_end, Try 2_| > | *I*_end, *T*__*ry* 4_| > | *I*_end, Try 3_|. This phenomenon is associated with the variability of the LRS and will be discussed later. Figure 4B highlights the principle of the SET probability, which would be 3 successful SET events divided by 4 attempted tries, resulting in a SET probability of 75% at the given voltage-time combination in this small scale example. Of course, a higher number of events is required for obtaining statistically significant ensembles.

Therefore, 15 individually contacted devices like the one shown in Figure 4A were tested experimentally for their SET probability traces. In this specific context, a SET probability trace is the probability-voltage relation that shows the required voltage range for a device to traverse from zero percent switching probability to 100% switching probability. The switching probability is determined analogously to the small scale example above. After the voltage stress, the resistance is determined. If it is lower than 20 kΩ, the device has undergone the SET process and the try is counted as successful. If the resistance is still above 20 kΩ, the try is counted as unsuccessful. The SET probability is then given as the fraction of successful events over the total amount of tries.

In this study, voltage stresses of 1 μs duration were employed. The chosen voltage range of -0.6 to -1.1 V with increments of -20 mV ensures that the entire trace is recorded with a sufficient resolution of the voltage range, where non-deterministic switching occurs, i.e., where the SET probability lies between 0 and 100%. At every voltage step, 50 tries are performed. This amount is a compromise between measurement speed and statistical significance. Hence, each given probability value has to be seen with an inaccuracy of at least 2%. Each try is proceeded by a forced SET, a RESET, and another SET using a sweep signal. The resistance state before the voltage stress is subsequently precisely programmed to be in the range of 200 and 350 kΩ before the SET try, which corresponds to read currents between -0.57 and -1 μA. The read currents immediately before pulse application are depicted as red histograms in Figure 4C (low current peak). The distribution lies well within the defined limits described above. Minor deviations at the lower and upper boundary are noticeable. This behavior has been studied before (Fantini et al., 2015; Wiefels et al., 2020) and can be explained by ionic noise that is typically present in filamentary VCM systems.

Figure 4D depicts both the measured and simulated SET traces of the described experiment. For better readability of the comparison, the gathered device traces are analyzed statistically in the following manner: The median trace value, as well as 5, 25, 75, and 90% percentiles at every tested voltage, are given. In this context, the term “edge cases” refers to the SET probability traces at the lower and upper voltage extreme. For the experimental dataset, the 5 and 95% essentially reflect the edge cases because of the limited device count. Therefore, the relative uncertainty in these percentiles is relatively large. For the simulation dataset, the actual edge cases may be located at slightly lower or higher voltages than the 5 and 95% lines, respectively. Here, the relative uncertainty is reduced because of the higher device number. While the agreement between simulation and experiment of the median and the 25 and 75% cases is nearly flawless, the compact model exhibits a slight mismatch for the 5 and 95% lines. However, this deviation is minor. The percentile lines are, however, an important characteristic for comparison to the simulation dataset. Overall, the experimental data shows the expected behavior of combined c2c and d2d variability. Following the median trace highlights the c2c aspect. It traverses from zero SET probability at low voltages to deterministic switching, i.e., a SET probability of 100%, at high voltages. The regime of non-deterministic switching has a width of around 160 mV. For the voltages in this range, the c2c variability leads to a mixture of successful and unsuccessful events. The percentile marks are indicative of the d2d spread of the devices. While the median trace of the experimental datasets shows the beginning of the non-deterministic regime at 0.80 V and the end at 0.96 V, the 25% trace is shifted to lower voltages of 0.70 and 0.86 V, respectively. The opposite trend is observed for the 75% trace which shows a range between 0.86 and 1.04 V. The range between these two traces, the interquartile range, is therefore almost perfectly constant at 160 mV for all voltages. The same observation can be made with the 5 and 95% traces. Here, a range of 280 mV is covered. These numbers are important measures for comparison to the simulation, but also comparison to other devices and device types. In cases where the SET event was successful, i.e., the resistance was below 20 kΩ, the resistance is noted. The red histogram at the higher current level in Figure 4C summarizes the read currents of the measured low resistance states. A significant spread from 10 μA up to 300 μA is visible. This spread further signifies the presence of variability in our devices. Three important points must be mentioned in this context: First, the displayed histogram is gathered from 15 different devices. Closer analysis reveals, that each device itself has a less significant spread. Hence, the d2d aspect of this measurement has to be taken into account. On top of that is the second point: The shown data summarizes both the d2d and the c2c variability of the devices. The third point is that in this measurement, low resistive states that result from SET pulses with varying amplitude are shown. In our previous work (Cüppers et al., 2019), we have demonstrated the impact of stronger voltages on the resistance state after the SET process. Therefore, voltage stresses that are barely enough to switch the device will lead to higher resistances than voltage stresses that switch the device with high certainty. Because of these three aspects, the spread of the low resistive state is not unexpected.

The simulation for this experiment was carried out with the model parameters described in section “Materials and Methods.” Two hundred fifty device seed parameters were drawn in the described way. Each drawn device is tested in the same way as described above for the experimental devices. Hence, the distributions of the HRS prior to the voltage stress and the LRS in cases where switching took place can be compared. Clearly, visible in Figure 4C is the nearly perfect agreement of the HRS distributions before the SET try. The comparison of the LRS values is more complicated. As visible from the comparison of distributions in Figure 4C, the simulation lacks parts of the distribution both at the lower current end and the higher current end. This difference is caused by two different phenomena. The lower current end divergence is caused by the imperfect description of the switching transition time by the model. As discussed in our previous work (Cüppers et al., 2019), the SET transition can be abruptly interrupted if the switching pulse is ended, but the device has not fully undergone the SET process. In the present model, the transition speed is higher than the experimental one, thus causing mainly full switching events. Therefore, the lower current end is not simulated as often as in the experiment. The higher current end divergence is due to the experimentally observed effect, that a stronger voltage leads to a higher read current, even after the switching event is completed. The employed simulation model cannot fully describe this relation, since in these simulations the defined maximum oxygen vacancy concentration is reached when the SET event takes place. In total, the switching model still describes the experimental data very accurately. The missing effects will be addressed in our future work.

As stated above, a much higher number of devices is simulated compared to the experimentally measured dataset. For the evaluation, the same approach as for the experimental dataset is chosen, and the median at each voltage as well as the 5, 25, 75, and 95% are calculated. The results are presented in Figure 4D. The good agreement for the median SET probability trace is visible. Moreover, all percentile traces are also well met, with only very minor deviations at the 5 and 95% traces.

In conclusion, the simulation of SET events on the 1 μs timescale reveals the high degree of agreement between measurement and simulation. All statistical values, including current levels and switching voltage, are well met. Slight deviations are caused by the inherent variability of both measurement and simulation on one hand and by minor effects not accounted for in the model on the other hand.

### Cycle-to-Cycle Switching Stochasticity Over Multiple Orders of Magnitude in Switching Time

To verify our simulation model beyond the correct c2c and d2d description on the 1 μs time scale, we test its predictive capability for a different experiment. A crucial prerequisite for a model is to correctly display the switching dynamics over multiple orders of magnitude in switching time (Menzel et al., 2011; Fleck et al., 2016; Cüppers et al., 2019; Bengel et al., 2020).

For this purpose, a single device was experimentally tested and the results were compared to the simulation. The chosen device follows the median trace of the 1 μs experiment very closely and is therefore considered as a good example. In this experiment, the device is switched by voltage pulses that vary over multiple orders of magnitude in pulse duration and voltage stress. The device preparation procedure for SET probability testing is identical to the one described in section “Device-to-Device Variability Characterization on 1 μs Timescale.” However, in this experiment, the device is stressed with a voltage pulse of variable duration (100 ms down to 100 ns) and amplitude (-400 to -925 mV in -25 mV decrements). The test procedure is repeated 25 times at each combination of pulse width and pulse amplitude.

**FIGURE 5:**
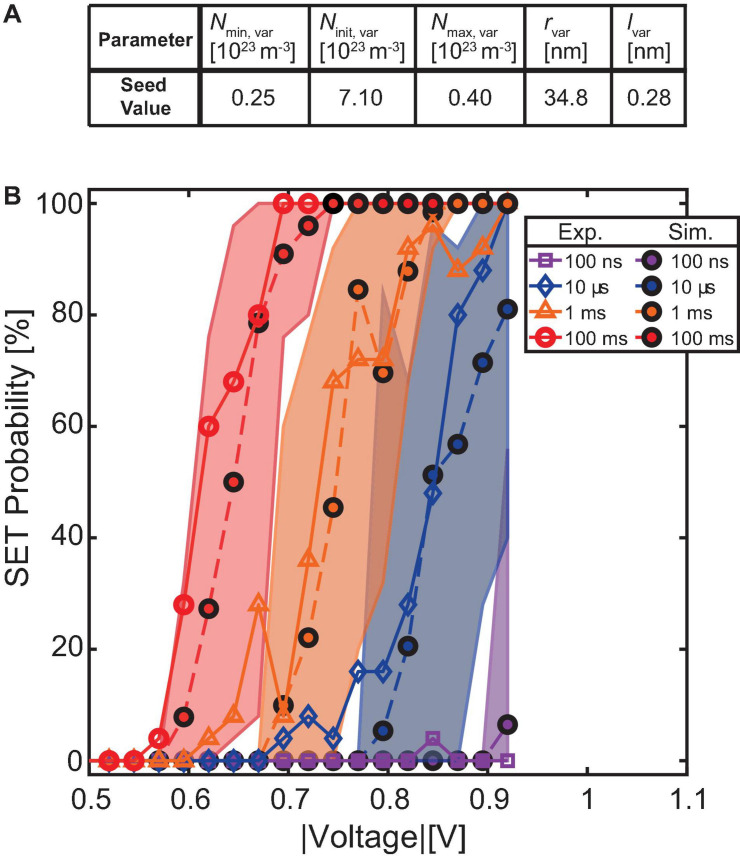
**(A)** Shows the seed parameters of the simulated device. **(B)** Shows the SET probability as a function of the pulse amplitude for different pulse widths. An experimental and a simulation device, which are both close to the median on the 1 μs timescale, are compared. Both voltage window and voltage onset in dependence of pulse duration are correctly described, verifying the predictive character of the compact model. The envelope areas quantify the maximum possible voltage shifts resulting from insufficient repetitions at a given voltage.

Figure 5 displays the experimental SET probabilities at each combination as a solid line. Noticeably, the onset of the SET probability curve shifts to lower voltages when increasing the pulse duration. This phenomenon has been studied before and reflects the typical SET switching kinetics (Fleck et al., 2016; Witzleben et al., 2017; Nishi et al., 2018; Cüppers et al., 2019; Bengel et al., 2020; Böttger et al., 2020). However, the voltage range, where the probability is neither 0 nor 100%, remains roughly constant at around 160 mV, with some deviations caused by the limited number of tries.

To reproduce this behavior over multiple orders of magnitude in switching time, a simulation identical to the described experiment was conducted by choosing a device seed parameter that follows the simulated median trace of the previously described 1 μs experiment. However, for this median-like simulation device, the pulse duration dependent SET probability traces are recorded not only once, but 50 times. Figure 5 contains the results of this simulation. Here, the dashed lines represent the SET probability at each combination of pulse duration and voltage stress, calculated from the 1,250 tries attempted at this combination. At the same time, the colored areas outline the range that is covered by each subset of 25 tries per combination. This method was chosen to highlight the fact, that a SET probability trace for the same device can show a shift of the voltages when recorded twice. The true SET probability trace is revealed by repeating the same pulse conditions a significant amount of times. In our experiment, 25 attempts already yielded stable results, but with each additional try, the accuracy of the SET probability trace can be increased.

In Figure 5, only every second pulse duration is shown for better readability. However, the not shown pulse durations follow the same trend of onset voltage and show the same width of the traces.

By comparing the experimental results to the simulation, it is directly visible that the median simulated curves closely follow the experimental ones apart from some minor deviations likely caused by the limited number of tries in the experiment. The key characteristic, namely the voltage range of non-deterministic switching, is met reasonably well.

The good agreement between measurement and simulation validates the JART VCM v1b switching model for this kind of experiment. The predictive character, namely that the compact model can describe the switching dynamics precisely on the 1 μs scale as well as for multiple orders of magnitude in pulse time, has been demonstrated. Therefore, we employ this model in the following sections for our pattern classification network.

### Experimental Demonstration of Parallel Operation

In this section, we describe the implementation of the proposed parallel synapse model from a theoretical standpoint and experimentally demonstrate the operation. Several devices are connected in parallel and are biased with identical voltage stresses of 1 μs duration.

For the proposed probabilistic update, it is desired to apply a voltage pulse of a given amplitude and get the probabilistic bitline current response as the outcome, like it is described in section “Network Fundamentals.” Therefore, different current response levels have to be accessible with some probability. By increasing (reducing) the voltage stress, the probability of getting a higher (lower) current response should increase. In the following, we will explain how ReRAM device variability affects the collective synapse behavior by using our compact model to simulate the different exemplary cases.

#### Device Requirements for Favorable Synapse Behavior

**FIGURE 6:**
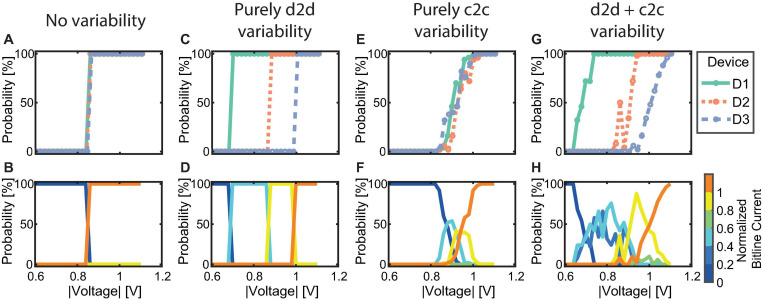
Simulation of different synapse behavior to showcase the relation between SET probability traces of individual devices (upper row) and probabilities for the achieved bitline currents (lower row) by adapting the compact model. **(A,B)** Represent three identical deterministically behaving devices. **(C,D)** Show three different but deterministic devices. **(E,F)** Show three identical but probabilistically behaving devices and **(G,H)** show three different and probabilistic devices.

For our proposed synapse implementation, it is required for the synapse to be able to adopt intermediate current levels between the two extreme cases of all devices in HRS and all devices in LRS. If all devices were to show identical, deterministic behavior, no intermediate current levels can be achieved, and the accumulated synapse current will either be low or high for low and high voltage signals, respectively. This undesirable or even worst-case scenario is illustrated in Figures 6A,B, where A shows the SET probability traces of three devices with identical seed parameters and no c2c variability, while B shows the probability for normalized bitline current levels at each voltage. As can be expected the synapse will only show two achievable current levels since the LRS and HRS values are the same for all three devices which switch in a deterministic fashion at a specific voltage.

The addition of d2d variability leads to a significant change in the synapse behavior. Figures 6C,D show three devices without c2c variability but with significant d2d variability. Different voltage onsets yield (*n*+ 1) separable bitline current levels, with *n* being the number of devices per synapse. Each of the levels has exactly 100% probability in a distinct voltage interval as the devices still show deterministic switching at a voltage specific to each device. The simulation was performed by modifying the seed parameters to achieve a device switching at a low, a moderately high and a high voltage. The device switching at a low (high) voltage is realized by choosing a small (large) filament radius (*r*_var_), a small (large) disc length (*l*_var_) and a high (low) initial oxygen vacancy concentration in the disc (*N*_disc, init_).

Adding c2c variability to situation A, instead of d2d variability leads to the behavior observed in Figures 6E,F. The devices shown here have the same parameter seed. Throughout the simulation, their parameters were varied to achieve c2c variability. At each voltage 50 tries were performed for each device. We would like to note here that this limited number of repetitions at each voltage is the reason for the three different SET probability traces. Increasing the number of repetitions will make the traces comparable and even identical for an infinite number of repetitions. Since we can only simulate a limited number of cycles, the SET probability traces are different. In Figure 6F, the resulting bitline current range probabilities form a voltage window. In this voltage window, the intermediate bitline current ranges can be addressed with a single voltage pulse. However, the voltage window has a width of only around 200 mV although the total range of applied voltages is 600 mV.

The previous cases are, however, theoretical cases since filamentary switching VCM type devices exhibit significant d2d and c2c variability as a consequence of the underlying physical mechanism as well as tolerances in device fabrication. This makes it virtually impossible to eliminate variability. By combining d2d and c2c variability, we arrive at the most realistic case shown in Figures 6G,H. Compared to the previous case, the voltage window is significantly wider (around 400 mV), which is caused by the early onset voltage of device D1 and the late onset of D3. In this specific case, it would be sufficient to employ voltage levels with a spacing of around 100 mV to address the intermediate bitline current ranges. Another feature of real devices that can be observed here is that the number of accessible levels is larger than (*n*+ 1). This can be attributed to the variability in the LRS state of the various devices as shown in Figure 4C. It should be noted here that similar to E and F resulting bitline current probabilities in H are not an unambiguous consequence of the SET probability traces in G due to the limited number of tries at each voltage level. Each try of all devices at a specific voltage can be viewed as a discrete stochastic process.

In summary, to realize an analog synapse with binary switching devices, the interplay between d2d and c2c variability is very important. Deterministic devices limit the number of levels to two while adding d2d leads to an increase of the number of levels in a deterministic fashion (the levels can be programmed with 100% probability). Adding c2c variability leads to probabilistic devices and a probabilistic update. The voltage window to achieve this, however, might be limited. Differing probabilistic devices widen this window, but are not necessarily required for the proper synapse functionality if their individual c2c variability covers a sufficiently wide voltage window. For an increasing number of devices per synapse, the range of conductances will shift toward higher values. However, if we scale this range by the number of devices in each synapse, we can see that the resulting fraction stays constant. Thus, the bounds to the synaptic efficacy stay constant except for a shift toward higher conductance levels, which might lead to a higher power consumption. On the other side, the analog tunability improves as more intermediate levels become accessible. Lastly, this improvement in the synapse behavior will increase the area that each synapse occupies. As d2d and c2c cannot be eliminated in real devices our observations need to be tested by measurements.

#### Experimental Demonstration

**FIGURE 7:**
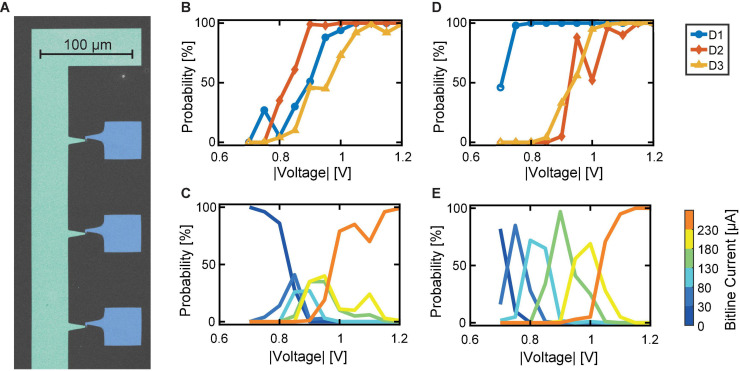
Experimental demonstration of favorable synapse characteristics. **(A)** SEM picture of a section of the device structure contacted in a probe card arrangement. Blue: Top electrodes. Green: Bitline electrode (color added in post-processing). **(B)** SET probability traces of three similar devices. **(C)** Resulting bitline current probabilities, showing the narrow voltage range for tunability. **(D)** SET probability traces of three significantly different devices. **(E)** Resulting separated bitline current ranges.

To verify our proposed explanation of the synapse behavior in dependence of the included variability, we show two exemplary cases of experimentally realized synapses. Therefore, we applied the voltage stress to three devices simultaneously. In order to do so, we employ a probe card arrangement as described in the experimental section “Probe Card Arrangement.” From the 32 × 1 line array, we contacted three devices to see the direct dependence of the device SET probability traces on the synapse characteristic. The structure is shown in the scanning electron microscopy (SEM) picture in Figure 7A. The addressed cases reflect the cases discussed in Figures 6G,H. In this experiment, the three devices in parallel were repeatedly stressed with voltages from -0.7 to -1.2 V in steps of -50 mV. Each voltage was tested 100 times. The initialization was carried out for each device individually and followed the same procedure as described in section “Device-to-Device Variability Characterization on 1 μs Timescale.” After each voltage stress, the summed bitline current is recorded for a read voltage of -0.2 V. On top of that, each device is read individually with a read voltage of -0.2 V.

First, we contact three devices with very similar SET probability traces, see Figure 7B. The resulting synapse behavior is depicted in Figure 7C. As expected, the bitline current ranges are only addressable in a very limited voltage range of about 0.2 V. This means, that intermediate synapse currents require very precise voltage stresses to the devices. This combination of devices reflects the case of identical probabilistic devices as described in Figures 6E,F. In contrast, the second subset of devices contacted shows a significant voltage margin between the individual SET probability traces, see Figure 7D. At 0.70 V, the highest probability is observed for the lowest current range of 0–30 μA, since only infrequently switching events occur and the devices remain in the HRS. A lower probability is evident for the second current range of 30–80 μA. By increasing the voltage to 0.75 V, the probability of reaching this second level is increased. However, the first level or the third level may occur with a low probability. This trend continues in a very regular pattern at 100 mV intervals until currents of 230 μA or more are observed. Here, the currents do not increase further with voltage because the synapse’s dynamic range is reached. Therefore, at 1.20 V, the probability for reaching the last level, i.e., currents above 230 μA, is 100%. The dynamic voltage window of this synapse, therefore, lies in the voltage range from 0.70 to 1.05 V, which results in a voltage window of 0.35 V. This behavior is favorable over the case of nearly identical devices, since a higher voltage spacing for the levels can be utilized, which in turn reduces the challenges for integration.

In summary, our proposed synapse structure fulfills the imposed requirements. The synapse’s tunability window is mainly influenced by the switching probability behavior of the individual devices it is composed of. Two possible ways for enlarging this window can be derived: First, the presence of d2d variability can improve the synapse behavior. However, only having d2d variability can still lead to unwanted synapse behavior if nearly identical devices happen to appear in a given synapse. A better approach is to introduce more c2c variability in each device while reducing the d2d variability to a minimum. By this method, the voltage window remains large enough for several voltage levels with moderate spacing. At the same time, the minimized d2d variability ensures conformality of the synapse characteristic.

#### Synapse Simulations

**FIGURE 8:**
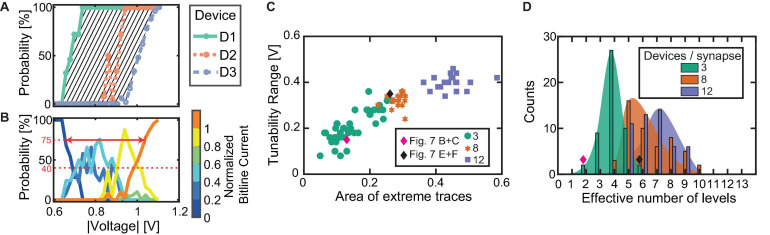
**(A,B)** Sketch of the introduced analysis parameters based on the individual SET probability traces and the bitline current probabilities. The gray shaded area in **(A)** shows the first parameter, while the solid red line in **(B)** shows the second and the dashed red line in **(B)** shows the third. **(C)** Dependence between tunability range and extreme trace area for the simulations comprising 50 instances of 3, 8, and 12 devices each. **(D)** Statistics of the effective number of addressable intermediate bitline current ranges for each synapse size.

For different synapse sizes, namely 3, 8, and 12 devices per synapse, we simulated 50 synapses. At each voltage, 50 SET tries were conducted. For comparing the arising effects when scaling up the synapses, we introduce three new parameters as indicated by the sketch in Figure 8A:

1. The difference between the first and the last SET probability trace with respect to the pulse voltage. For this, the area between the two extreme traces is calculated by the trapezoidal numerical integration of the difference of the first and last trace, see the shaded area in Figure 8A.
2. The synapse tunability voltage range. It is calculated by the difference in voltage of the first current range at a probability of 75% and the voltage of the last current range at a probability of 75%. This value describes the range of voltages in which the intermediate current ranges (between “all devices HRS” and “all devices LRS”) can be stochastically addressed. It should be adequately large for the chosen voltage levels of the application. The value is marked with an arrow in Figure 8B.
3. The effective number of realistically addressable levels. For this, the number of levels that reach a probability of 40% or more over the whole voltage range is counted. It is highlighted by the horizontal dashed line in Figure 8B.

Figure 8C displays the relation between the SET probability difference and the synapse tunability voltage range for all synapse sizes and each individually initialized synapse. The simulations with three devices per synapse are indicated by green circles. The achievable voltage window for a synapse constructed from three devices ranges from quite low values to the desired larger ranges. For three devices per synapse, an increasing relation of the tunability range with the area enveloped by the highest and lowest SET trace is obtained. The exact underlying relation is, however, masked by the significant variability, which stems from the combination of variable devices. The simulation results are controlled by the experimental data. For this purpose, the desired data points are determined from measurements shown in Figures 7B,C and in Figures 7D,E, respectively, and are added to the graph in Figure 8C as diamond-shaped symbols. The data points from the experiment lie within the range of the simulated multi-device synapses for the case of three devices. This match clearly demonstrates the accuracy of the developed compact model.

A further enlarged voltage tunability range of a multi-device synapse can be achieved by increasing the number of devices per synapse. Figure 8C also displays the simulation results for 8 and 12 devices per synapse. For the purpose of comparing these synapses, the bitline current ranges were normalized with the assumption that each device contributes 38 μA (corresponds to 5.2 kΩ) to the overall bitline current. Furthermore, instead of splitting the resulting currents into 6 levels as for the 3 devices per synapse, the currents are grouped in 11 and 15 levels for 8 and 12 devices per synapse, respectively. For the 8 and 12 devices per synapse structures, the tunability window is above 0.2 V, and levels at around 0.4 V. The synapse comprising 12 devices shows an even higher SET probability difference, but no significant increase in the tunability window is evident. Most important for synapses comprising 8 and 12 devices is the absence of synapses with an undesirable low voltage tunability window. This can be attributed to the low chance of drawing 8 or 12 nearly identical devices for the synapse, respectively. By increasing the synapse size, it becomes increasingly likely to draw devices from the full d2d range, hence making sure that sufficient variability is present in the synapse. For the three devices, there is a chance of getting three highly similar devices with their limited c2c voltage range, thus causing low voltage window synapses as e.g., shown experimentally in Figures 7B,C. Figure 8D shows the statistical analysis for the 50 simulated synapses regarding the effective number of addressable intermediate bitline current levels for each synapse. As expected, the number of levels with a probability of 40% or more increases with synapse size, allowing for more accurate tuning of the larger synapses. The experimentally determined data points are plotted as diamond symbols. Again, the data points derived for the extreme cases lie at the edges of the simulated range. It is therefore expected that increasing synapse size will yield higher network performances, especially if overlapping patterns are shown since such require improved synapse tunability.

### Classification of Overlapping Spatio-Temporal Patterns

The general simulation procedure for network simulation is described in section “Network Fundamentals.” While during the training phase the network’s weights are adjusted to minimize the pattern recognition error, the network also contains hyperparameters. Hyperparameters are these parameters used to control the learning process. Unlike the weights, the hyperparameters are not derived through training but have to be specified prior to the training. For our utilized network, the hyperparameters are defined by the learning rate λ, the neuron threshold current *I*_TH_, the maximum achievable spike rate *FRMAX*, which also determines the spike decision threshold, and the SET and RESET voltages for the synapses. While these parameters have a significant impact on the network performance, they are typically not trained but rather preset to a constant value. Therefore, their choice has to be motivated in a different way, which usually involves trial and error. In this work, however, we describe a plausible procedure to tune two of the hyperparameters, namely *I*_TH_ and *FRMAX*, before the training of the neural network starts. This alleviates the problem of finding optimal hyperparameters, since two parameters less have to be optimized manually.

#### Tuning of the Hyperparameters

ReRAMs exhibit significant d2d and c2c variability as discussed in the previous sections. This makes finding global parameters for the neuron threshold (*I*_TH_) and the decision threshold (*FRMAX*/2), which ensure good network convergence, challenging. In any case, a global combination of *I*_TH_ and *FRMAX* for all neurons would be a compromise and would degrade the performance by having this global constraint. Our approach is to mitigate this issue at a local scale by self-adapting the values at the individual neuron level. Before training the network, we first strongly excite the neuron by turning all synapses on, using a stronger SET pulse (-1.3 V for 1 μs) to achieve a global SET probability of 100%. We then sample the output current that is achieved for several different exemplary *pattern+* training signals (applied to 11 random synapses). We need to excite all synapses because of the external noise (flipped bits). By varying *I*_TH_, we can sample the maximum possible number of spikes that can be generated by this output neuron for different *I*_TH_. In a second step, all synapses are turned off by applying a RESET pulse (+1.3 V for 1 μs) and the output current is sampled for several different exemplary *pattern-* training patterns. By varying *I*_TH_, we can then sample the minimum possible number of spikes that can be generated by this output neuron for different *I*_TH_. By averaging over the responses, we can find the value of *I*_TH_ that results in the largest difference between the neurons response to *pattern+* and *pattern-*. With this *I*_TH_ the decision threshold *FRMAX*/2 is defined as the difference between the weakest response of the neuron to the different *pattern+* signals and the strongest response of the neuron to the different *pattern-* signals. It is found that the optimum *I*_TH_ is increased with the number of ReRAM cells per synapse as a higher current will pass through the parallel devices. The decision threshold *FRMAX*/2 is found more or less independent of the number of devices per synapse. The values are distributed between 20 and 30 Hz. The tuning algorithm is formalized in Algorithm 2. In summary, the proposed algorithm maximizes the distance between the neurons response for *pattern+* and *pattern-*. An additional advantage is that it enables adaptation to failed devices and even would enable retraining of the network. This might be useful if after a certain time some of the devices start to fail. In that case, the described procedure can be repeated and adapted hyperparameters can be found that consider the failed devices. After these hyperparameters have been algorithmically optimized, the training starts.

**Table:** **Algorithm 2**: Hyperparameter tuning algorithm.

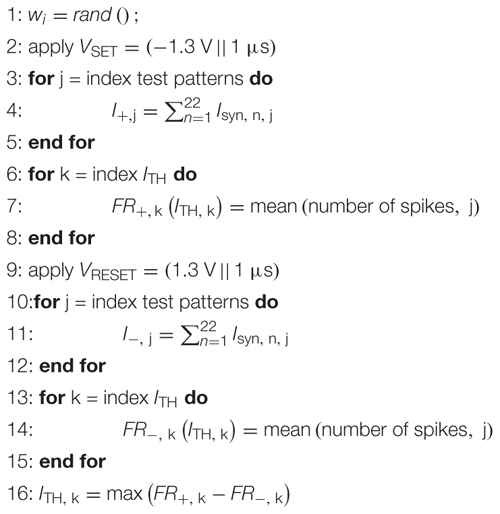

#### Neural Network Performance in the Presence of Overlapping Patterns

**FIGURE 9:**
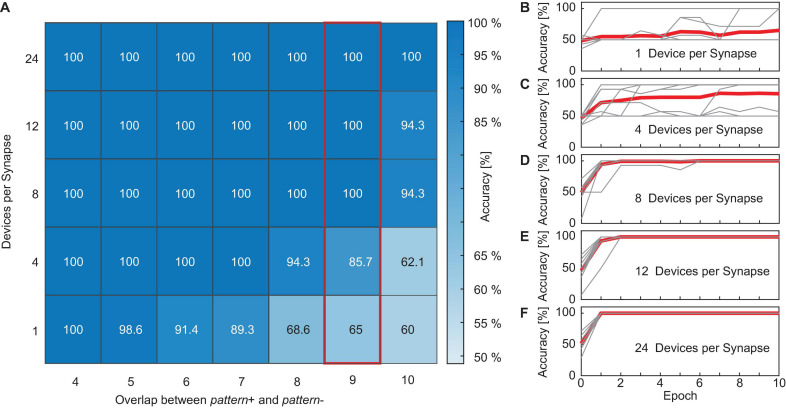
**(A)** Simulation results of the neural network’s accuracy as a function of the overlap between the patterns and the number of devices per synapse. **(B–F)** Show the accuracy over the training epochs for all 10 runs (thin gray lines) as well as the mean curve (thick red line) for an overlap between the patterns of nine. **(B)** Shows the results if each of the 22 synapses consists of only one ReRAM device, **(C–F)** for 4, 8, 12, and 24 devices per synapse, respectively.

First, we looked at how the achievable accuracy of the neural network changes if the overlap between *pattern+* and *pattern-* is increased and how the accuracy is improved if the number of devices per synapse is increased. The studied ranges for the overlap were 4, 5, 6, 7, 8, 9, and 10 while the studied range of devices per synapse was 1, 4, 8, 12, and 24 devices. Figure 9A shows the simulation results of the neural network’s accuracy as a function of the overlap between the patterns and the number of devices per synapse. If the accuracy reached 100% after a certain training epoch, the training was stopped and this accuracy was taken as the final value of this run. Otherwise, the accuracy after 10 epochs was used. Figures 9B–F show the evolution of the accuracy over the training epochs for all 10 runs (thin gray lines) as well as the mean curve (thick red line) for an overlap between the patterns of nine. B shows the results if each of the 22 synapses consists of only 1 ReRAM device, and C to F for 4, 8, 12, and 24 devices per synapse, respectively. From this figure, multiple effects can be observed. Due to the different sources of variability that already exist in the initialization phase of the network (d2d, Poisson inputs, etc.), 10 runs were performed for each combination of overlap between the patterns and number of devices per synapse. From Figure 9A it can be observed that the overlap between the two patterns influences the network’s performance. We generally observed that the network reliably reaches an accuracy of 100% for small overlaps (<5), independent of the number of devices per synapse. For larger overlaps, the average accuracy is degraded. However, this effect is stronger for networks where only a small number of devices are used. This shows that increasing the number of devices per synapse is a way to improve the performance of the neural network if the classification problem becomes more difficult. This can also be observed in Figures 9B–F. While networks with only one or four devices per synapse struggle to reach an accuracy of 100% during training, perfect accuracy can be achieved after only one training epoch for networks with more devices. While some runs also achieve high accuracies after a few training rounds for the small networks, other runs struggle as their accuracy is stuck at a low value or oscillates over the epochs.

**FIGURE 10:**
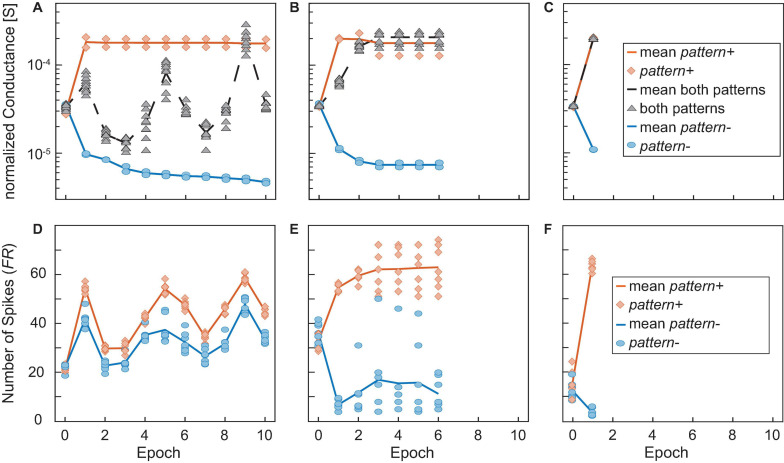
**(A–C)** Show the conductances normalized to the number of devices per synapse of the synapses receiving *pattern+* (orange line and diamonds), *pattern-* (blue line and circles) and both patterns (black line and triangles) over the training epochs. The overlap was 9 in all cases and the number of devices per synapse was 1 in **(A,D)**, 8 in **(B,E)**, and 24 in **(C,F)**. The solid and dashed lines show the mean values while the different symbols show the values of the actual synapses. **(D,E)** and **(F)** show the corresponding numbers of spikes (*FR*) that are generated if *pattern+* (orange) or *pattern-* (blue) is presented to the neuron. The lines again show the mean values while the symbols show the number of spikes generated for the unique patterns.

A closer look at the training is depicted in Figure 10. Here, the conductances of the different categories of synapses for the runs in Figures 9B,D,F as well as the number of spikes that were generated for positive and negative patterns are shown. A, B, and C show the conductances normalized to the number of 1, 8, and 24 devices per synapse, respectively, of the synapses receiving *pattern+* (orange line and diamonds), *pattern-* (blue line and circles) and both patterns (black line and triangles) over the training epochs. Again, the overlap between the patterns was nine. The solid and dashed lines show the mean values while the different symbols show the values of the actual synapses. D, E, and F show the corresponding number of spikes (*FR*) that are generated if *pattern+* (orange) or *pattern-* (blue) is presented to the neuron. The lines again show the mean values while the symbols show the number of spikes generated for the unique patterns. Similarly to Figure 9, we observe that a higher number of devices improves the network’s performance. While A and D (one device per synapse) show that the training is not complete after 10 epochs the training already finishes after the sixth epoch for B and E (eight devices per synapse) or after the first training epoch for C and F (24 devices per synapse). Looking closer at Figure 10A shows the reason why the training is not successful. While the synapses receiving only *pattern+* (orange) or only *pattern-* (blue) are programmed to distinct values that stay constant throughout the training, the synapses receiving both patterns (black) are not programmed to a stable conductance level and change throughout the training, oscillating between the conductance boundaries. Since the overlap was nine in this example, the group of synapses receiving exclusively *pattern+* or *pattern-* each only consists of two elements while the black group consists of nine elements. The consequences of this can be seen in Figure 10D, which shows the number of spikes generated in this case for *pattern+* (orange) *pattern-* (blue). The distance between the neuron’s responses to *pattern+* and *pattern-* is small and both are subject to abrupt changes. This prevents a converging of the delta rule algorithm as the training voltages are not scaled down. A contrast to this can be seen in Figures 10B,E which were achieved for an increased number of devices per synapse of eight. In this case, the synapses receiving both patterns quickly reach a stable conductance range. As can be expected the neuron responds to this increase (decrease) in current with a significantly higher (lower) number of spikes for *pattern+* (*pattern-*). Lastly, C and F show an even improved picture. The training is already finished after the first training epoch as all synapses achieve a stable conductance value. It can be observed in A, B, and C that the neural network only reaches 100% accuracy, when the synapses receiving both patterns (black) are completely excited. Our explanation for this finding is that fully excited and fully depressed synapses are representative or more stable device states in the sense that they require higher voltages to be switched. It has been shown that a higher HRS requires higher SET voltages to set the device and that a smaller LRS requires higher RESET voltages to reset it. Therefore, if a synapse is found in a fully excited or depressed state, it will require higher absolute voltages to switch it to the opposite state as if the synapse was only partially excited or depressed.

In summary, our results show that increasing the number of devices per synapse greatly increases the performance of the network as it allows for a more gradual tuning of the weights and helps with reaching the stop learning condition (100% accuracy).

### Neural Network Stability for Noisy Input Patterns

**FIGURE 11:**
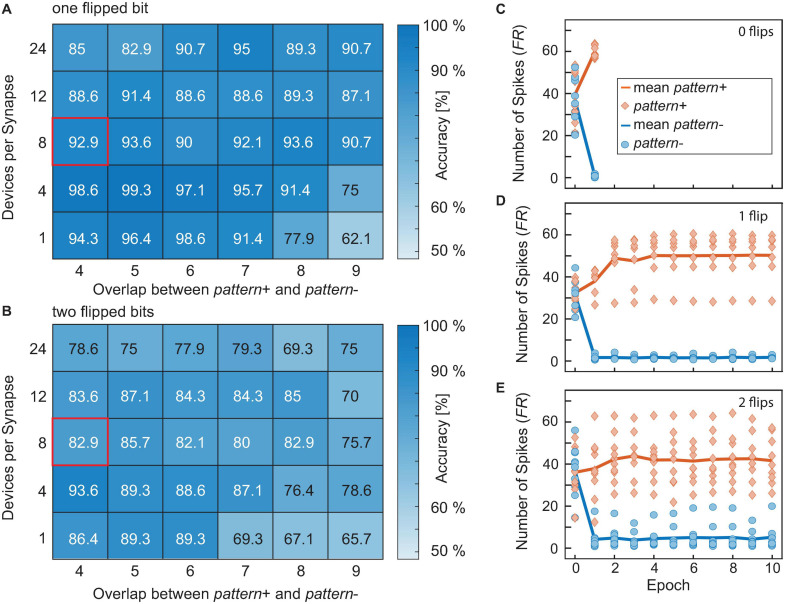
**(A,B)** Show the accuracies achieved for one **(A)** or two flipped bits **(B)**, respectively, averaged over 10 runs evaluated after the same criterion that was used in Figure 9. **(C–E)** Show the number of spikes generated by *pattern+* (orange) and *pattern-* (blue). The solid lines show the median while the diamonds or circles show the responses to the unique *pattern+* or *pattern*-, respectively. **(C)** Shows the results for zero flips, **(D)** for one flip and E for two flips.

As the last example, we look at the network’s performance when the inputs are noisy. The number of flipped bits represents an external noise source. For the case of *N* flipped bits, *N* random synapse assignments are changed for every training and test pattern. This makes the classification problem significantly more difficult since causes the wrong synapses being trained. Here a range of zero flipped bits up to two flipped bits was tested. For higher numbers of flipped bits, the accuracy is heavily degraded and no network architecture was able to reliably achieve accuracies much higher than random guessing. An overview of the results for one and two flipped bits can be seen in Figures 11A,B. As expected, the accuracy worsens when some of the input bits are flipped in each training and test run. Another important feature to be observed in Figures 11C–E is that the unique runs showcased by the orange diamonds and the blue circles show a significantly larger spread if the number of flips is increased, resembling the significant rise in pattern to pattern variability. The different test patterns are fixed before the training starts and they are not changed over the epochs. While some patterns produce more easily distinguishable spike numbers (close to zero for *pattern-* or about 60 for *pattern+*), other patterns provide not such a clear spike response. The number of patterns producing an unclear response increases with the number of flipped input bits. For zero flips all patterns provide a clear spike response, for one flip there is one pattern that falls out of line and for two flips most of the patterns provide an unclear spike response. This degradation can also be seen in the median spike response for *pattern+*, which is close to 60 for zero flips, around 50 for one flip and only at 40 for two flips.

**FIGURE 12:**
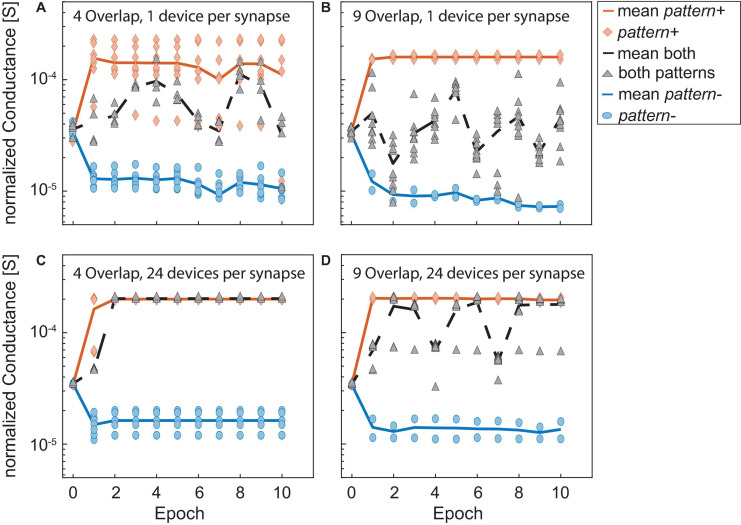
**(A–D)** Show the conductances normalized to the number of devices per synapse of the synapses receiving *pattern+* (orange line and diamonds), *pattern-* (blue line and circles) and both patterns (black line and gray triangles) over the training epochs. The solid and dashed lines show the mean values while the different symbols show the values of the actual synapses.

The dependence of the accuracy on the number of devices per synapse and the overlap between the patterns is, however, more complicated than before. One trend which can be observed is that for smaller overlaps the smaller networks usually perform better than their larger counterparts. Our explanation for this is that when the network becomes larger it stops training the weights after the first few epochs as the error-adjusted SET and RESET voltages become too small to significantly adjust the weights. Figures 12A,C show this exemplarily for the case of two flips, an overlap of 4 and the networks containing one (A) or 24 (C) devices per synapse. The conductances of the synapses in Figure 12A are much more shallow than their counterparts in Figure 12C, which enables the network to reach a better final result. The synapses in Figure 12C show very little change after around the second epoch which means that the network has stopped training at this point. As we saw in Figure 10 larger networks generally lead to a faster convergence, as they can find weight values for high accuracies quicker. In the presence of flipped bits in the inputs, this behavior will still hold. However, as some of the synapses are trained in the opposite direction, this initial stable solution does not yield 100% accuracy. Their smaller counterparts take longer to find a stable solution as the weights are easier disturbed. This gives the smaller synapses a certain robustness against the incidence of flipped bits. An increase in overlap stresses the point that larger networks perform generally better for one flip, see Figure 11A. For two flips the same statement is true, see Figure 11B.

Multiple effects determine the final accuracy of a network with a given size and overlap that can be achieved. On the one hand, smaller networks have an advantage if the overlap between the patterns is small or medium as their less stable weights can be tuned even if the error becomes smaller (see Figures 12A,C). However, larger networks perform better for one flipped input and larger overlaps, which can be explained in the same way as for the zero flip cases. The comparison of the smallest and largest considered network for an overlap of nine and two flipped input bits in Figures 12B,D shows why larger networks can find better solutions for high overlaps than smaller networks. While the unique synapses receiving both patterns (gray triangles) for the small network are mostly programmed to a less stable medium conductance states (see Figure 12B), the larger network can program them to a more stable high conducting state (see Figure 12D).

Overall, the accuracy is reduced if the inputs are noisy. As can be expected, this effect is stronger if more inputs are noisy. As the drop in accuracy is closely related to the utilized training rule, it seems reasonable to investigate how to increase noise resilience. As seen from the device analysis, this noise resilience will have to take into account the specialties of ReRAM programming. One idea toward this might be to use a non-linear scaling of the voltage in the delta rule algorithm. If—for the sake of argument—we assumed that the first spike response was at a maximum distance from the goal spike response, this would leave us with a *Δw*_i_ of 1 (see Eq. 2), giving the nominal programming voltage *V*_SET_ or *V*_RESET_ in the training step. If the next spike response to an applied pattern was now at half the distance from the goal spike response, the resulting *Δw*_i_ would be 0.5 corresponding to half the SET or RESET voltage to be applied afterward. This dividing of the training voltage in half is, however, problematic when applied to our devices. As can be seen in Figure 4D, the range of voltages that lead to a 50% SET probability is around 300 mV, considering also the edge cases. This range is not significantly changed if one goes to other SET probabilities. If now the nominal SET voltage was -1 V, to ensure a 100% probability, half of that would be -500 mV, which would result in no switching at all. For half the algorithmic response the actual response is more or less set to zero. Of course, this assumed case does not occur frequently, and typically our voltage scaling approach works. Also the SET probabilities are affected by the HRS with smaller HRS states leading to an increased probability for a constant voltage. Still, there is room for improvement if adapted scaling of the training voltages is performed.

## Discussion

Here, we reported on a synapse concept that employs multiple filamentary VCM-type memristive devices in a parallel configuration. The goal was to achieve the favorable multilevel conductance tuning required in the majority of neuromorphic hardware architectures. To this end, the switching stochasticity, which is an inherent feature of these devices, was exploited. The following points summarize the main findings of this study:

First of all, it has become obvious that for a correct device description it is necessary to study multiple devices on multiple timescales and with high repetition numbers. Findings based on single device experiments or limited cycle numbers are to be considered with extreme caution, as inaccurate c2c and d2d variability assumptions can change the results on higher levels of integration architectures significantly. Primarily, this can be seen in the shown examples in Figure 6 and in Figure 1. Various combinations of c2c and d2d variability lead to different synapse behavior. If not modeled with respect to a minimum statistical range of devices, wrong conclusions on the synapse behavior might be drawn, leading to suboptimal operation, which will increase the mismatch between simulation and experimental investigation of a network.

Second, it is crucial to evaluate the agreement between experimental results and the employed simulation tool in detail as shown in this work. Several aspects need to be addressed accurately:

• Resistance distributions of HRS and LRS
• SET voltage onset and distribution
• Switching dynamics
• Device-to-device spread

Ultimately, simulation tools like the proposed one are unavoidable for testing novel neuromorphic concepts for their feasibility in real-world situations. In this context, accurate device compact models may be seen as an important step on the way toward large-scale neuromorphic applications, just like transistor models are at the foundation of current processing units.

Third, we investigated the concept of parallel devices for a single synapse. Our bottom-up derivation of favorable synapse behavior concluded that for our desired application it is very important to have a certain amount of variability in total, which may be composed of both c2c and d2d variability to different extents. This minimum variability in the synapse composition translates into favorable tunability of the synaptic weight. We showed that additional devices in the synapse compound enhance this tunability factor, hence enhancing the synapse performance. We attributed this effect to an increased number of realistically addressable levels, a larger operation voltage window and more distinct levels per synapse. For this purpose, we introduced three parameters to assess the quality of a compound synapse, verified through experiment and simulation.

An interesting outlook for the future presents itself. In more advanced integration routes the amount of d2d will most likely be reduced, while the c2c amount will remain as it is a consequence of the physical nature of the VCM-type resistance change. Therefore, the addressed case with small d2d variability may arise. We consider our approach to be resilient toward this development, as the requirement lies within the interplay of c2c and d2d variability. However, adjustments regarding the operation voltages may become necessary because the voltage window is significantly reduced under these circumstances, requiring a voltage spacing in the tens of millivolts in our case.

Fourth, we integrated the proposed synapse structure into an exemplary neural network which was trained using a technologically plausible algorithm making use of the concept of stochastic rounding. By developing an optimized hyperparameter tuning scheme for our devices, the network was able to converge to 100% accuracy for easy tasks. As expected from the previous discussion, higher complexity problems, i.e., higher overlap between the patterns, required additional devices per synapse to maintain high accuracy. Here, a top-down view on the network training stage revealed that a higher device count per synapse leads to more resilience against perturbations in the form of pattern overlap. However, this stability proved to have a weakness when additionally considering input noise, i.e., flipped bits, in the training stage. As the final synapse weight was reached after a single epoch for large synapses, noise in the form of flipped bits leads to degraded accuracies. In contrast, fewer devices per synapse required multiple epochs for reaching the minimum error, therefore averaging over multiple flipped bit events. Hence, for low overlaps, a lower device count surpassed the performance of higher device numbers per synapse, while high overlap tasks were better solved by higher device count synapses. One mitigation strategy of this unexpected result may present itself in a more conservative voltage scaling approach, which begins at a lower voltage and employs smaller voltage increments. By this technique, the prolonged learning stage allows averaging over multiple flipped bit patterns and therefore adds noise robustness to the network. The need for adjustments like the developed hyperparameter tuning algorithm and the device-aware network operation emphasizes the importance of algorithms that are tailored to the physical substrates.

As memristive devices have become widely used in neuromorphic applications in recent years, the concept of using multiple devices per synapse has been applied to different realizations of memristive devices. Examples for experimental realizations of this concept were done for Electro-Chemical Metallization (ECM) cells (Gaba et al., 2013) and Phase Change Mechanism (PCM) cells (Boybat et al., 2018), as well as other VCM systems. In addition, since the primary requirement for employing the concept is switching voltage variability, we consider it to be applicable to other VCM systems such as Ta_2_O_5_ (Nishi et al., 2018), TiO_2_ (Yoon et al., 2013), SrTiO_3_ (Rieck et al., 2021). However, in many systems, the possibility of analog switching has been demonstrated. Further studies are required for a parallel configuration of such analog type switches since our concept is based on digital switches with two distinguishable states.

However, the diverse resistance switching phenomena observed in these systems will require careful design of the synapse operation algorithms. For instance, higher resistance variabilities will reduce the realistic number of addressable synapse current levels, while a tighter switching voltage distribution may reduce the voltage window where conductance tunability is possible. The parameters derived in the present study are able to capture these device-related characteristics and offer comparable quantities for different devices and device types.

On the network level, mainly theoretical results have been obtained so far due to the difficulty of large scale integration possibilities.

Singha et al. (2014) used simulations to investigate this synapse concept for showing Spike Timing Dependent Plasticity (STDP) behavior. Their findings showcase that increasing the number of parallel devices in the synapse brings the synapse closer to the optimal analog case. However, in their study, they did not consider resistance variability nor d2d variability. At the current state of memristive device research, these two issues have not been resolved, but may be reduced in the future. Our modeling therefore represents a more realistic picture of the current state of the art. Even including the described artifacts, we were able to achieve promising results, suggesting that the concept can compensate for some of the perceived device shortcomings. Also, they did not go to the network level to investigate the performance of a neural network based on their synapses.

Bill and Legenstein (2014) proved the feasibility of the proposed synapse concept in a STDP update rule from a theoretical point of view and with simulations of idealized bistable devices. Their study came to the similar conclusion, that the network classification error can be reduced by increasing the synapse resolution, i.e., increasing the number of devices per synapse M. However, in their abstract model, they did not consider conductance variability in the states, leading to the assumption that each synapse can assume up to M + 1 discrete conductance. A more realistic case is shown in our study, where the actual addressable number of states per synapse is lower than M + 1, caused by the conductance variability. However, the overall trend of performance gain is maintained, which is in line with their study. Furthermore, the study predicts a strong resilience of parallel device synapses against device non-uniformity, which we can confirm from our study.

Overall, the results obtained from previous literature studies and our study agree that the proposed concept of multiple devices per synapse is a promising approach, presenting a feasible alternative to single analog devices as synapse elements. However, by introducing multiple devices per synapse, new challenges arise due to the device characteristics, which were shown to have direct impact on synapse levels and tunability window. Moreover, multiple devices per synapse results in an increased area footprint and peripheral CMOS circuitries. Solutions such as the demonstrated hyperparameter algorithm will be required to access the full potential of this promising approach.

## Conclusion

An extensive study of the c2c and d2d variability phenomenon was presented. An adaptation of our highly detailed physics-based compact model allowed us to study the neuromorphic concept of employing multiple parallel devices as synapse. Through experiment and simulation utilizing the compact model, we verified the feasibility of the concept of analog synapse tuning by single voltage pulse operation. It was found that by increasing the number of devices per synapse, a larger voltage window and a higher number of realistically addressable current ranges are realized in the synapse. This is explained through the interplay of c2c and d2d variability of the devices. The proposed concept fundamentally requires at least one degree of variability, hence it will also work in the presence of reduced d2d variability in future improved fabrication routes. The synapse structure was then tested in a pattern classification SNN, for which we developed a device inspired novel hyperparameter tuning algorithm that considers synapse inhomogeneities. As predicted from the study on single synapses, the network’s ability to detect overlapping patterns is improved by increasing the number of devices per synapse, which is attributed to the higher degree of incremental tuning capability. It was found that in the presence of noise, a slower convergence to the final network state is favorable, which can be realized by adapted voltage scaling algorithms in the proposed concept. This study highlights the importance of physically derived simulation models for the evaluation of neuromorphic concepts.

## Data Availability Statement

The original contributions presented in the study are included in the article/supplementary material, further inquiries can be directed to the corresponding author/s.

## Author Contributions

FC conducted the experiments. CB performed the modeling and simulation. MP contributed to the theory. SH-E, SM, MP, RD, and RW performed the review, editing, and supervision. FC and CB wrote the manuscript equally. All authors listed have made a substantial, direct and intellectual contribution to the work, and approved it for publication.

## Conflict of Interest

The authors declare that the research was conducted in the absence of any commercial or financial relationships that could be construed as a potential conflict of interest.
